# Boosting Vaccine-Elicited Respiratory Mucosal and Systemic COVID-19 Immunity in Mice With the Oral *Lactobacillus plantarum*

**DOI:** 10.3389/fnut.2021.789242

**Published:** 2021-12-22

**Authors:** Jianqing Xu, Zhihong Ren, Kangli Cao, Xianping Li, Jing Yang, Xuelian Luo, Lingyan Zhu, Xiangwei Wang, Longfei Ding, Junrong Liang, Dong Jin, Tingting Yuan, Lianfeng Li, Jianguo Xu

**Affiliations:** ^1^Zhongshan Hospital, Institutes of Biomedical Sciences, Shanghai Medical College, Fudan University, Shanghai, China; ^2^Shanghai Public Health Clinical Center, Fudan University, Shanghai, China; ^3^State Key Laboratory for Infectious Disease Prevention and Control, National Institute for Communicable Disease Control and Prevention, Chinese Center for Disease Control and Prevention, Research Units of Discovery of Unknown Bacteria and Function (2018 RU010), Chinese Academy of Medical Sciences, Beijing, China; ^4^Institute of Public Health, Nankai University, Tianjing, China

**Keywords:** COVID-19, vaccine, probiotics, adjuvant, gut-lung axis, memory immunity, *Lactobacillus plantarum*, accquired immunity

## Abstract

Boosting and prolonging SARS-CoV-2 vaccine-elicited immunity is paramount for containing the COVID-19 pandemic, which wanes substantially within months after vaccination. Here we demonstrate that the unique strain of probiotic *Lactobacillus plantarum* GUANKE (LPG) could promote SARS-CoV-2-specific immune responses in both effective and memory phases through enhancing interferon signaling and suppressing apoptotic and inflammatory pathways. Interestingly, oral LPG administration promoted SARS-CoV-2 neutralization antibodies even 6 months after immunization. Furthermore, when LPG was given immediately after SARS-CoV-2 vaccine inoculation, specific neutralization antibodies could be boosted >8-fold in bronchoalveolar lavage (BAL) and >2-fold in sera, T-cell responses were persistent and stable for a prolonged period both in BAL and the spleen. Transcriptional analyses showed that oral application of LPG mobilized immune responses in the mucosal and systemic compartments; in particular, gut-spleen and gut-lung immune axes were observed. These results suggest that LPG could be applied in combination with SARS-CoV-2 vaccines to boost and prolong both the effective and memory immune responses in mucosal and systemic compartments, thereby improving the efficacy of SARS-CoV-2 vaccination.

## Introduction

The continued circulation of severe acute respiratory syndrome coronavirus 2 (SARS-CoV-2) has greatly impacted the global public health system and resulted in more than 249 million cases and over 5 million deaths. Hopes for returning to normal have largely depended on scaling-up inoculation with effective vaccines that are essential to prevent further morbidity and mortality. To date, there are 194 and 128 COVID-19 vaccine candidates in preclinical or preclinical development, respectively, and 21 vaccines are currently been offered to the general population (1). The World Health Organization estimated that at least 70% of the world's population will need to be vaccinated to stop the pandemic. The persistent evolution of SARS-CoV-2 led to the emergence of new contagious variants that fueled the spread of disease. It remains unknown how effective the vaccines are against these mutations and how long they will protect against viral transmission (2). The antibody responses following natural infection wane substantially within months (3), so it is possible that immunity generated by vaccines might only last for months. Thus, containing the pandemic will require strategies that boost and prolong vaccine-elicited immune responses.

Probiotics confer a health benefit to the host when administered in adequate amounts (4). We hypothesize that oral administration of certain specific strain of probiotics could enhance and probably prolong COVID-19 vaccine-elicited immune responses through a mechanism of regulating both innate and acquired immune responses. Here we demonstrate that a unique strain of probiotic *Lactobacillus plantarum* GUANKE (LPG) is capable of promoting SARS-CoV-2 specific immune responses in both effective and memory phases through enhancing interferon (IFN) signaling and suppressing apoptotic and inflammatory pathways.

## Materials and Methods

### Ethics Statement and Animals

The aim of this study was to determine the effect of LPG on the immune response to the SARS-CoV-2 vaccine in mice. All animal-related procedures were conducted according to the protocol approved by the Institutional Animal Care and Use Committee (IACUC) of Shanghai Public Health Clinical Center (Shanghai, China). Specific pathogen-free (SPF) female ICR and BALB/c mice were purchased from Shanghai Jihui Biological Co., Ltd and housed in the animal facility at Shanghai Public Health Clinical Center in environmentally controlled cages with a 12-h-light/-dark cycle under SPF conditions. Mice were provided with free access to water and diets.

### Mice Immunization

The aim of the first experiment was to determine the effect of oral LPG administration on mouse humoral immune responses to COVID-19 when given 6 months after immunization. All ICR mice (*n* = 10) received intramuscular vaccine injections at weeks 0 and 4 post priming, and sera were collected between weeks 5 and 24 post priming for antibody assessment by enzyme-linked immunosorbent assays (ELISAs) and pseudovirus inhibition assays. At 24 weeks post vaccination, the mice were given water containing 1 g/L of ampicillin for 5 days to prevent colonization resistance (5), then they were randomly divided into two groups that received oral administration of either 200 μL of LPG (5 × 10^9^ CFU, *n* = 6) or phosphate-buffered saline (PBS, *n* = 4) once daily for 3 days. The antibody titers measured immediately before ampicillin administration were set as the baseline to be compared with measurements taken at various time points post LPG or PBS administration. Sera were collected on day 7, 14, 28, and 42 post oral administration of LPG or PBS for receptor-binding domain (RBD) binding antibody assessment by ELISA and neutralization antibody (nAb) quantification by pseudovirus inhibition assay.

The second experiment was conducted to determine the effect of oral LPG administration on humoral and cellular immune responses in mice when given immediately after immunization. All ICR mice were primed with an intramuscular injection of 100 μg of DNA-S at week 0. At week 12 after priming, the mice were given drinking water containing 1 g/L ampicillin for 5 days then boosted with 1 × 10^11^ viral particles (vp) of AdC68-CoV/Flu via both intramuscular and intranasal routes. The mice were then randomly divided into two groups (*n* = 9/group) for oral administration of either 200 μL of LPG (5 × 10^9^ CFU) or PBS once daily for 3 days. The antibody titers measured immediately before ampicillin administration were set as the baseline to be compared with measurements taken at various time points post LPG or PBS administration. The mice (*n* = 3/group) were sacrificed on day 3, 7, 14 post administration of LPG or PBS. Sera and bronchoalveolar lavage (BAL) were collected on baseline and day 7 post administration of LPG or PBS for RBD binding antibody and nAb assessment. To assess RBD-specific T cell responses, splenocytes and BAL cells were isolated on day 3, 7, and 14 post oral administration of LPG or PBS and *in vitro* stimulated with 13 peptide pools (15-mer with 11 overlapped amino acids) covering the entire RBD sequence. The resulting IFN-γ secreting cells were quantified by ELISpot. Interleukin (IL)-6 and tumor necrosis factor (TNF)-α in BAL from mice sacrificed on day 3, 7, and 14 were quantified using Th1/2/17 Assay Kit (Cat#560485, BD Bioscience, Franklin Lakes, NJ, USA).

### Oral LPG Administration of Mice for Transcriptional Analyses

To examine how LPG imprints the immune system. Two groups of ICR mice (20 mice per group) were given 1 g/L of ampicillin for 5 days followed by oral administration of 200 μL of LPG (5 × 10^9^ CFU) or PBS once daily for 3 days. Different tissues including spleen, mediastinal lymph node (LN), mesenteric LN, small intestine, and colon tissues were collected and stored in TRIzol at different time points after oral administration for RNA-sequencing.

### Bacterial Culture

LPG isolated from the feces of healthy people was routinely grown in Man-Rogosa-Sharpe medium in a 37°C carbon dioxide incubator (Forma CO2, Thermo Fisher Scientific, Waltham, MA, USA). The logarithmic phase of LPG was washed and resuspended with sterile PBS for oral inoculation of mice.

### Cell Lines and Viruses

The human cell lines used in this study included HEK 293A and HEK 293T. All cell lines were purchased from American Type Culture Collection (Manassas, VA, USA) and maintained in complete Dulbecco's modified Eagle's medium supplemented with 10% fetal bovine serum (FBS) and 1% penicillin-streptomycin (PS) (D10) at 37°C in a 5% CO_2_ incubator. The generation of wild-type SARS-CoV-2 pseudovirus was described in our previous publications (6, 7). In brief, HEK 293T cells were co-transfected with pNL4-3.Luc.R-E- (cat#3418, NIH AIDS Reagent Program) and pcDNA3 plasmid encoding SARS-CoV2-S using TurboFect reagent (cat#R0531, Thermo Fisher Scientific). At 24 h post transfection, the culture medium was refreshed with D10 medium for an additional 48-h incubation. The pseudovirus-containing supernatants were harvested by centrifugation then filtered through 0.45-μm filters and stored at −80°C as single-use aliquots. For each pseudovirus stock, a titration assay was performed using an aliquot to determine the dilution needed for the neutralization assay.

### DNA Vaccine Construction

The DNA-S is a DNA vaccine candidate for which the codon-optimized DNA sequences encoding SARS-CoV-2 spike (S) protein (YP_009724390.1) were synthesized (Generay Biotech Co., Ltd., Shanghai, China), and cloned into the pcDNA3.1 vector to generate the pcDNA3.1-S plasmid.

### Construction, Rescue, Propagation, and Purification of Recombinant Chimpanzee Adenoviruses

The AdC68-CoV/Flu is a chimpanzee adenoviral vaccine with a hemagglutinin stalk derived from influenza A/Shanghai/4664T/2013 strain and the RBD of wild-type SARS-CoV-2, which were synthesized and cloned into the E1 site of the recombinant AdC68 vector. The recombinant AdC68 is different from the wild-type AdC68 in that both the E1 and E3 genes are deleted. The E1 locus is essential for viral replication, so recombinant AdC68 viruses can only be produced in cell lines engineered to supply the E1 protein, such as HEK 293A. HEK 293A cells were seeded in 6-well plates for 24 h then were transfected with a PacI linearized corresponding AdC68 construct using Lipofectamine 2000 following the manufacturer's instruction (Invitrogen, Carlsbad, CA, USA). The rescued viruses were propagated in HEK 293A cells and purified using CsCl gradient ultracentrifugation in combination with desalting with Bio-Gel P-6 DG Media (Bio-Rad, Hercules, CA, USA).

### RBD Binding Antibody Detection Using ELISA

RBD binding antibody titers in serum and BAL samples were measured by ELISA. In brief, sera and BAL samples were heat-inactivated at 56°C for 30 min before use. Next, 96-well ELISA plates were coated with 100 μL of 1 mg/mL recombinant SARS-CoV-2 RBD protein (Z03483-1, Genscript, Piscataway, NJ, USA) at 4°C overnight. After washing the plates with 300 μL PBS containing 0.5% Tween-20 (PBS-T) and blocking with 200 μL 5% non-fat milk in PBST (PBST/5% milk) for 2 h at room temperature (RT), a 2-fold dilution series (generally starting from 1:100) of mouse serum and BAL samples were added, followed by 3-h incubation at RT. For immunoglobulin G (IgG) measurement, a 1:5,000 dilution of horseradish peroxidase (HRP)-conjugated goat anti-mouse IgG (ZB-5305, Zsbio, Beijing, China) was applied in 100 μL PBST/5% milk. After 1 h incubation at RT, the plates were extensively washed with 300 μL PBS-T before addition of the substrate OPD (one SIGMAFAST OPD tablet [SLCC0308, Sigma, St. Louis, MO, USA] in 20 mL of deionized water). The reactions lasted for 5 min at RT and were terminated by adding 1M H_2_SO_4_, followed by reading at OD492 with a Synergy Microplate Reader (Bio-Tek, Winooski, VT, USA). ELISA endpoint titers were defined as the highest dilution that yielded an absorbance that was 2-fold greater than the background value.

### Total IgG ELISA

Total serum IgG levels were measured using the IgG (Total) Mouse Uncoated ELISA kit (Cat# 88-50400, Thermo Fisher Scientific). Each sample was diluted 500-fold in assay buffer and run in duplicate with Southern Biotech TMB Stop Solution (Cat#0412-01) as the stop solution. Optical density values were measured at 450 nm and 570 nm on a VersaMax plate reader (Molecular Devices, Sunnyvale, CA, USA) and corrected by subtracting the measurement at 570 nm from the measurement at 450 nm. A four-parameter standard curve was used to calculate sample concentration values.

### Pseudovirus Neutralization Assay

The protocol for SARS-CoV-2 pseudovirus generation was described previously (7). For assessment of serum neutralizing activity against SARS-CoV-2, serial 50-μL dilutions of heat-inactivated serum were made with D10 medium and mixed with equal volumes of diluted pseudovirus. After incubation at 37°C for 1 h, the serum-pseudovirus mixtures were transferred to a 96-well plate containing hACE2-293T cells, which were pre-seeded at 2 × 10^4^ cells per well and allowed to grow for 12 h. The plates were incubated for 48 h at 37°C in the presence of 5% CO_2_, and luciferase activities were then measured using Bright-Glo™ Luciferase Assay System (Promega, Madison, WI, USA) on a luminometer (Promega GloMax 96). The neutralizing (50% inhibitory doses) titers were derived from the highest dilution resulting in a 50% reduction in relative light units (RLUs) relative to virus control wells after background (RLUs of no virus wells) subtraction.

### IFN-γ Based ELISpot Assay

T cell responses were analyzed by using single-cell suspension of splenocytes and BAL by mouse IFN-γ ELISpot assay set (Cat#551083, BD Bioscience) following the manufacturer's protocol. In brief, 96-well ELISpot plates were pre-coated with anti-mouse IFN-γ antibodies (5 μg/mL, 100 μL) overnight at 4°C. After rinsing with RPMI-1640 medium (10–040-CVR, Corning, Corning, NY, USA) containing 10% FBS (BI, 04–001-1acs) and 1% PS (30–002-CI, Corning) (R10), plates were blocked with R10 for 2 h at RT before adding to each well 2 × 10^5^ of splenocytes or 2 × 10^4^ of BAL cells, followed by stimulation with the indicated 15-mer peptide(s) or pool(s). The peptides used for the stimulation work were synthesized in ChinaPeptides Co., Ltd. Each assay was performed in duplicate. Peptide stimulation lasted for 20 h after plates were placed in a humidified 5% CO_2_ incubator at 37°C. The plates were washed before biotinylated anti-mouse IFN-γ antibody (2 μg/mL, 100 μL) was added for 2-h RT incubation. Next, streptavidin-conjugated HRP was added at 1:100 dilution and incubated at RT for 1 h. The plates were washed again and subjected to spot development with AEC substrate reagent (Cat#551951, BD Bioscience). The reaction was stopped by water rinsing, and the plates subsequently allowed to dry for 24 h in the dark. Plate images were captured with a Biospot plate reader (ChampSpot III, Beijing SageCreation Science Co., Ltd., Beijing, China) and analyzed for spot-forming cell counts. Quality control was implemented on the automatic counting data by removing pseudo-spots, such as those resulted from pollutants and filaments and other residues. R10 was used as a negative control and all the data were adjusted by subtracting the value of negative control, 50 SFCs/million was considered as cut off value.

### BAL Cytokines

Flow cytometry assessments of serum levels of IL-6 and TNF-α were carried out using the BD Mouse Cytometric Bead Array kit for Th1/Th2/Th17 cytokine panel (Cat#560485, BD Bioscience).

### RNA-Sequencing and Quantitative Real-Time Polymerase Chain Reaction

RNA-sequencing was performed to examine how LPG imprints the immune system. Total RNA of different tissues were extracted using the TRIzol agent following the manufacturer's instruction, and total RNA quality was determined using the Agilent 2100 (Agilent Technologies, Santa Clara, CA, USA). A total of 5 ng purified RNA was used for library construction with an Illumina® TruSeq® RNA Sample Preparation Kit v2 (Cat#RS-122-2001, Illumina, San Diego, CA, USA), and the library was sequenced on Illumina HiSeqTM2500 by Shanghai South Gene Technology Co., Ltd. (Shanghai, China). The clean data were obtained by removing adapter-containing reads and low-quality reads by FASTX-Toolkit (http://hannonlab.cshl.edu/fastx_toolkit/). These clean reads were mapped to the reference genes using Bowtie2 software and to the reference genome using HISAT software. Counts were converted to fragments per kilobase of exon per million mapped fragments (FPKM) values for normalization. Gene expression data were subjected to set enrichment analysis using gene set enrichment analysis (GSEA) and hierarchical cluster analysis with Multiexperiment Viewer (MeV 4.9).

### Statistical Analysis

All continuous variables are presented as means ± standard deviation. Univariate analysis of variance was used to compare the means of different groups, and Bonferroni or Dunnett tests, if appropriate, were used for multiple comparisons if their variance homogeneity was assumed. Otherwise, Kruskal-Wallis tests were used followed by pairwise comparisons if appropriate. Statistical analysis was performed using GraphPad Prism v.8.0 (GraphPad Inc., San Diego, CA, USA). Comparisons of antibody titer levels between the two groups were analyzed using unpaired *t*-tests or Mann-Whitney tests.

## Results

### LPG Promotes and Prolongs SARS-CoV-2 Specific Memory Antibody Responses in Mice

**Figure 1:**
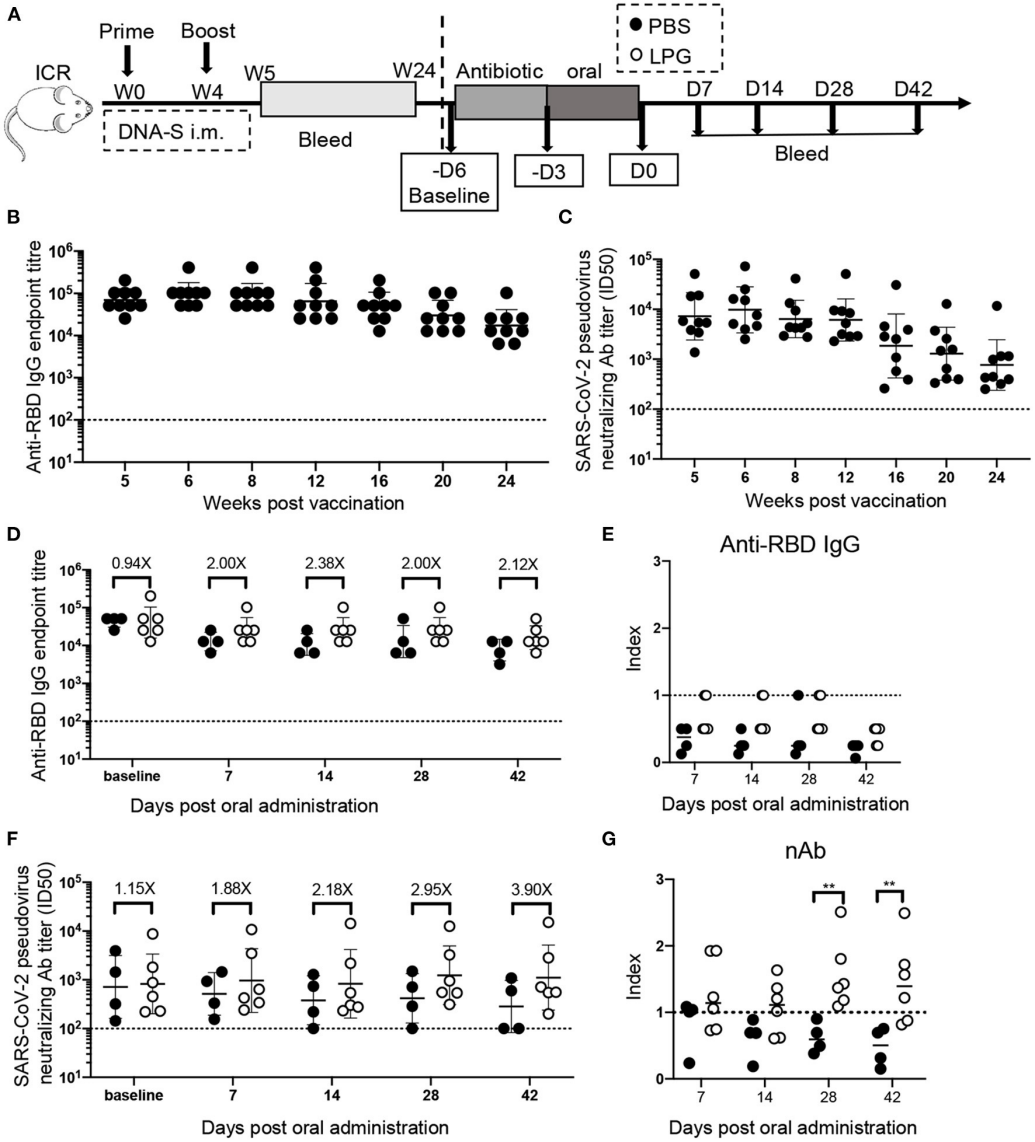
Oral LPG administration 6 months after immunization improved humoral immune responses in mice. Experimental schedule **(A)**: ICR mice were vaccinated with DNA vaccine at weeks 0 and 4, their binding **(B)** and neutralization **(C)** antibody responses were monitored by ELISA and pseudovirus inhibition assays respectively, from 5 weeks to 24, these mice were then orally administrated with PBS or LPG and observed for 42 days. The antibody titers immediately before antibiotic treatment were set as baseline. Sera RBD binding **(D)** and neutralization **(F)** antibodies on day 7, 14, 28, and 42 post administration of LPG or PBS were assessed by ELISA and pseudovirus inhibition assay, respectively. “Index” on the Y-axis represents the ratios of titers of binding antibodies **(E)** and neutralization **(G)** at indicated time points on the X-axis to their counterparts at baseline and reflects the adjusted relative values after the removal of baseline differences. D: Day; W: Week. Titer data are presented as geometric mean titer ± geometric standard deviation. Mann-Whitney tests were performed to analyze differences between experimental groups **P < 0.01.

In 1998, we accidentally found that oral LPG administration could boost the specific humoral immune responses against *Shigella dysenteriae* in vaccinated BALB/c mice (8). The unique strain of LPG we used is phylogenetically close to *L. plantarum* J26 with 12 additional genes based on whole genome sequences analyses of 611 strains available in public database (9) (Supplementary Figure 1). In this study, we sought to determine if LPG could also improve immune responses in a SARS-CoV-2 vaccinated model. A group of ICR mice were inoculated with the DNA vaccine at weeks 0 and 4 (Figure 1A). The results of the sera RBD binding antibody titers (Figure 1B) and nAb titers (Figure 1C) from weeks 5 to 24 showed that both antibody titers maintained their peak levels from weeks 5 to 8 and then decreased gradually over the 24-week follow up. Then these mice were orally administered with PBS or LPG and observed for 42 days. As expected, both groups had similar levels of RBD-specific antibodies and nAbs at baseline. The binding antibodies and nAbs gradually decreased in the PBS group (Figures 1D,F, filled circles), consequently, the index (ratios of titers of antibody at indicated time points to their counterparts at baseline, reflecting the adjusted values after the removal of differences at baseline) values decreased below one and further subsided over time (Figures 1E,G, filled circles). In contrast, the binding antibodies stabilized in the LPG group for at least 28 days (Figures 1D,E, open circles), while nAbs progressively elevated. Accordingly, the differences (folds between LPG and PBS groups in Figure 1F) also increased from 1.15 to 3.9 fold, which were further evidenced by the “index” values and were significantly different on day 28 and 42 (*P* <0.01; Figure 1G). To determine whether nAb enhancement was due to the overall increase of host total antibody responses, we compared total IgG titers and found no significant difference between the PBS and LPG groups (Supplementary Figures 2A,B). Together, these results demonstrate that LPG was able to boost and prolong memory antibody responses in the absence of vaccine or immunogen.

### Oral LPG Administration Immediately After SARS-CoV-2 Vaccination Significantly Boosts Both Effective Humoral and Cellular Responses Against COVID-19 in Mice

**Figure 2:**
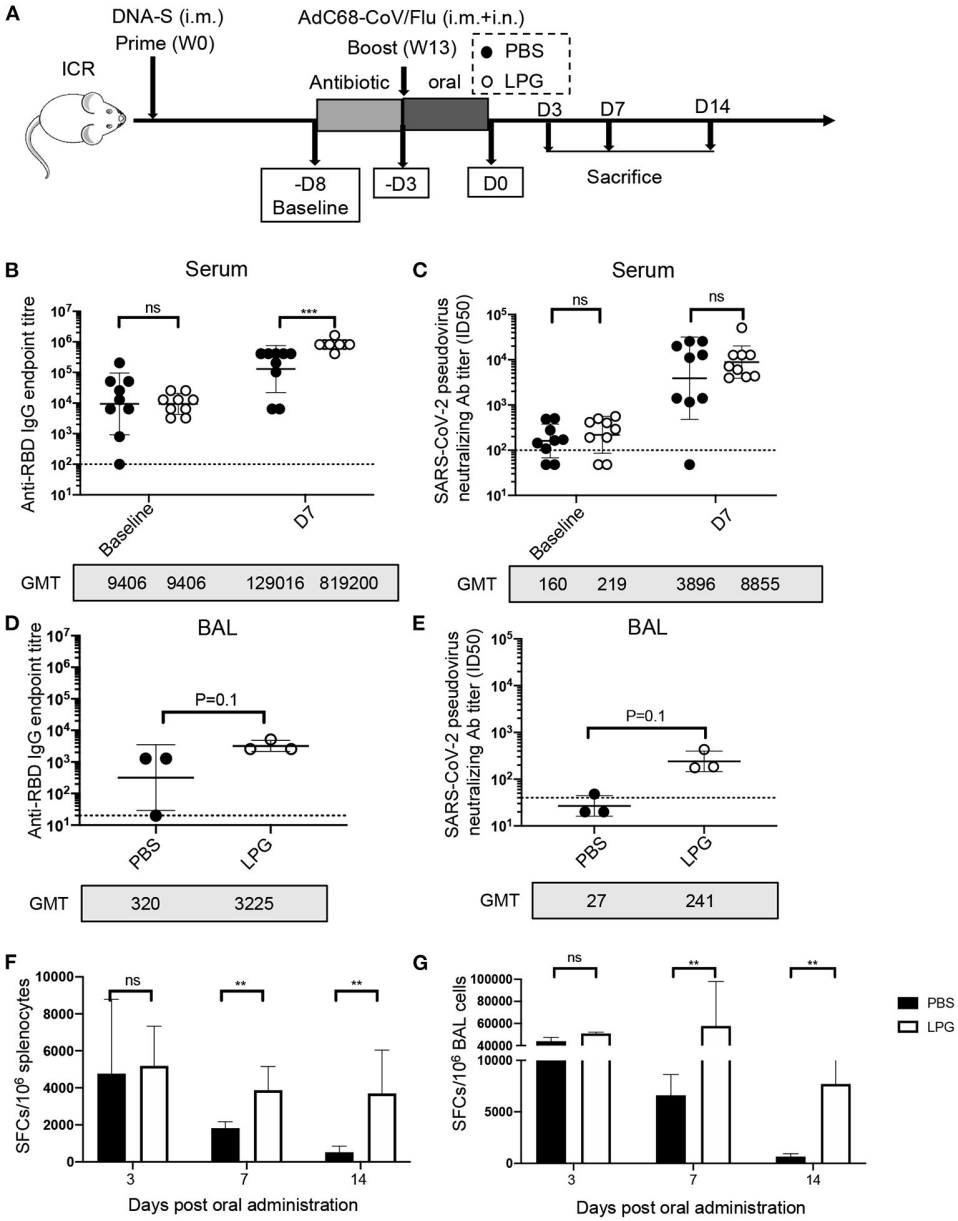
Oral LPG administration immediately after immunization improved humoral and cellular immune responses in mice. Experimental schedule **(A)**. ICR mice were primed and boosted with vaccines followed by oral administration of LPG or PBS as described in the Methods. Serum antibody responses were assessed at baseline (immediately before antibiotic treatment) and day 7 post intragastric administration (D7) for RBD-specific binding antibodies by ELISA **(B)** and pseudovirus neutralization assays **(C)**. BAL antibody responses were assessed on day 7 post intragastric administration for RBD-specific binding antibodies by ELISA **(D)** and pseudovirus neutralization assays **(E)**. Assessments of RBD-specific T cell responses. T-cell responses in splenocytes **(F)** and BAL cells **(G)** were determined on day 3, 7, and 14 post intragastric administration. BAL: Bronchoalveolar lavage; D: Day; W: Week. Titer data are presented as geometric mean titer ± geometric standard deviation; ELISpot counts were expressed as mean ± s.e.m. Mann-Whitney tests were performed to analyze differences between experimental groups. **P < 0.01, ***P < 0.001, and ns, not significant.

We next sought to determine whether LPG was capable of boosting vaccine-induced effective immune responses in the SARS-CoV-2 vaccination model. ICR mice were primed and boosted with vaccines followed by oral administration of LPG or PBS as described in detail in the Methods (Figure 2A). As shown in Figure 2B, serum RBD binding antibody titers at baseline (before ampicillin treatment) were comparable in the PBS and LPG groups with a geometric mean titer (GMT) of 9,406 (left panel). On day 7 after boost, the titers increased to GMT 129,016 in the PBS group, and a further 6.3-fold increase to GMT 819,200 was observed in the LPG group (*P* < 0.001; right panel). Similarly, a 2.3-fold promotion of nAb responses was observed in the LPG group compared with the PBS group, although significance was not reached (Figure 2C, right panel). We further assessed binding antibody and nAb titers in BAL collected on day 7 post oral administration. Binding antibody titers in the LPG group (GMT = 3,225) were ~10-fold higher than in the PBS group (GMT = 320; Figure 2D), as were nAb titers in the LPG group (GMT = 241) vs. the PBS group (GMT = 27; Figure 2E). We next examined RBD-specific T cell responses in the spleen and in BAL isolated from immunized mice on day 3, 7, and 14 post oral LPG administration by ELISpot assay, which captures IFN-γ after stimulation with pools of RBD-derived peptides. A total of 13 RBD peptide pools were used to fully cover the RBD sequence, each comprising five peptides (15-amino acid) that overlap by 11 amino acids. Although the boost vaccination induced similar T-cell responses in the LPG and PBS groups (data shown as day 3), they rapidly decreased in the PBS group and remained relatively stable in the LPG group (Figures 2F,G and Supplementary Figures 3A,B). Collectively, these results suggest that oral LPG administration boosts effective humoral and cellular responses against SARS-CoV-2 in vaccinated mice.

### Oral LPG Regulates Immune Responses Through Gut-Lung and Gut-Spleen Immune Axes

**Figure 3:**
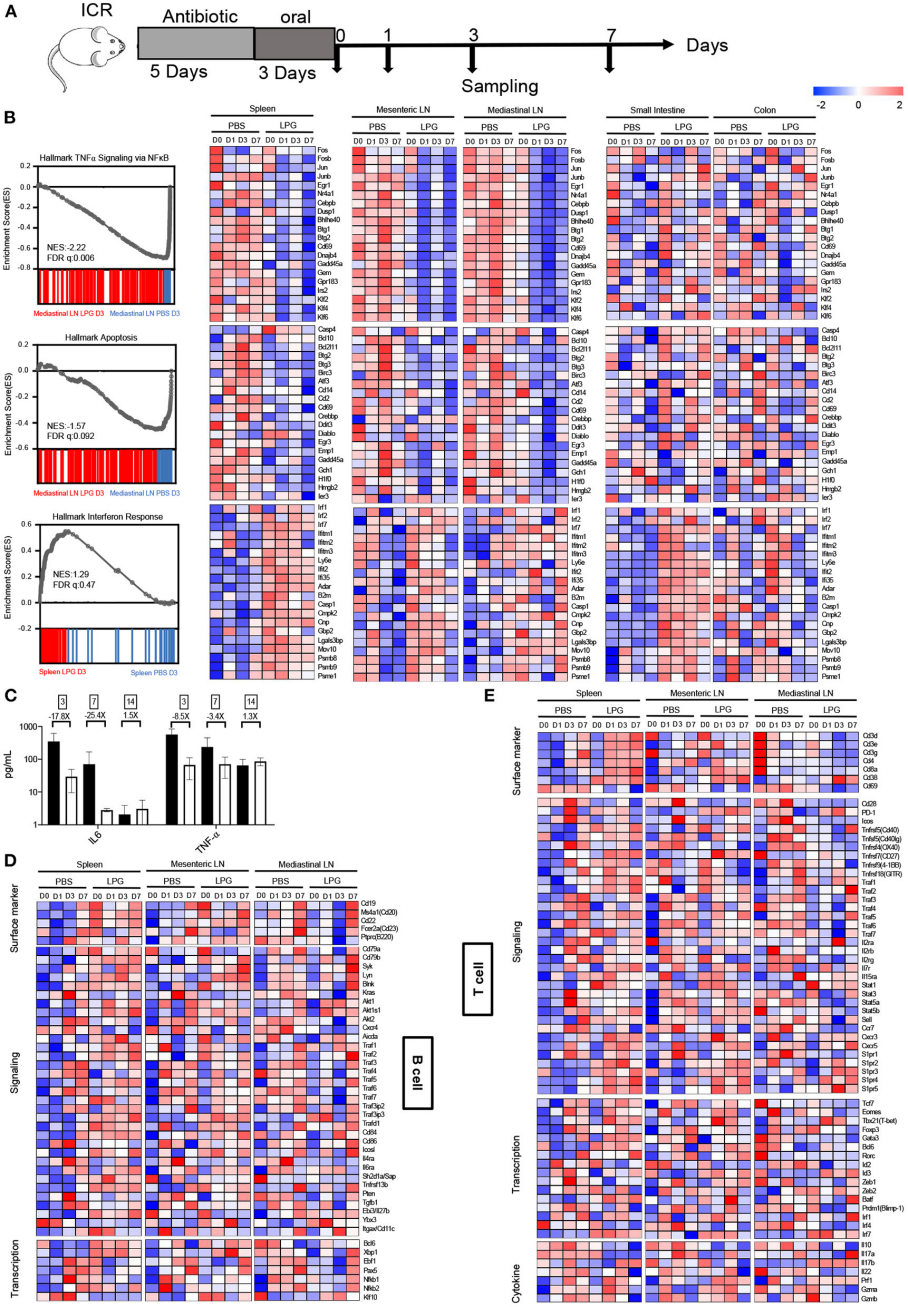
Oral LPG administration down-regulates inflammation and apoptosis but promotes IFN signaling in mouse lymphoid tissues. Experimental schedule **(A)**. ICR mice (*n* = 20/group) were treated as described in the Methods, and different tissues were collected for RNA sequencing. GSEA revealed inhibition of TNF-α signaling (top) and apoptosis (middle) gene sets in lymphoid tissues in the LPG group compared with the PBS group, and enhancement of IFN response (bottom) gene sets in lymphoid and small intestine tissues in the LPG group compared with the PBS group **(B)**. IL-6 and TNF-α in BAL were quantified from mice sacrificed on day 3, 7, and 14 **(C)**. Heatmap shows log2 RPKM values of a selection of genes associated with B cells **(D)** and T cells **(E)** from the spleen and small intestine. BAL, bronchoalveolar lavage; ES, enrichment score; FDR-q, false discovery rate q value; NES, normalized enrichment score.

To examine how LPG imprints the immune system, we collected spleen, mediastinal LN, mesenteric LN, small intestine, and colon tissues to define their transcriptional landscapes with RNA-sequencing after oral LPG administration (Figure 3A). Surprisingly, three similar transcript profiles were identified among mesenteric LN, mediastinal LN, and spleen, including downregulated TNF-α (a representative marker of inflammation), apoptosis, and enhanced IFN responses in the LPG group compared to the PBS group (Figure 3B), suggesting that oral LPG can exert effects in both the mucosal and systemic immune systems to promote immune cell activation and differentiation. Interestingly, LPG actively down-regulated the TNF-α pathway, as evidenced by reduced expression of AP-1 transcription factor family members (*FOS, FOSB, JUN, JUNB)* (10) (Figure 3B, upper panel). It also dampened apoptosis pathways, shown by decreased expression of the apoptotic genes *BCL2L11, BTG2*, and *BTG3* (11, 12) (Figure 3B, middle panel). This down-regulation began on day 0 and peaked on day 1 for the mesenteric LN and spleen and on day 3 for the mediastinal LN, indicating that LPG almost simultaneously exerted its effects on the mesenteric LN and spleen with a slight delay for the mediastinal LN. However, this down-regulation was not observed in small intestine and colon tissues, suggesting that this mechanism largely functions on immune cells rather than digestive tissues. To further corroborate this observation, we quantified IL-6 and TNF-α in BAL from mice sacrificed on day 3, 7, and 14. A ~20-fold reduction in IL-6 and 3-8-fold decrease in TNF-α were observed on day 3 and 7, respectively, in the LPG group compared with the PBS group (Figure 3C).

In contrast to down-regulation of TNF-α and apoptosis signaling, LPG up-regulated the IFN response pathway as manifested by increased expression of IFN-regulatory factor 7 (*IRF7)* and IFN-stimulated genes *IFITM1/2/3* (13) in the small intestine, spleen, mesenteric LN, and less for the mediastinal LN, but not for colon tissue (Figure 3B, down panel). This up-regulation was triggered in both digestive tissues and lymph tissues with the exception of colon tissue. These results indicate that oral LPG administration can affect the lung immune response through the gut-lung and gut-spleen immune axes.

### Oral LPG Administration Promotes T and B Cell Responses Through Costimulatory and Survival Pathways

We next examined how the above-described immune microenvironment influences adaptive immune responses. A heatmap with hierarchical clustering analyses of RNA-sequencing on the T cell- and B cell-associated genes showed different transcriptional signatures in lymphoid and intestine tissues (Supplementary Figure 4), suggesting that the datasets are reliable. As shown in Figure 3D, a number of genes in the B-cell response (BCR) signaling pathway were up-regulated in the spleen and mesenteric LN on day 0 in the LPG group. Delayed up-regulation of those genes was also observed on day 7 in the mediastinal LNs. In addition, the co-stimulatory molecules *CD86, ICOSL*, and TNFR-associated factor (*TRAF*) family members (14) for B cell activation, proliferation, and differentiation were enhanced in the LPG group. Furthermore, several genes associated with B cell functions such as *AKT1/2, AICDA*, and *EBI3* were upregulated in the LPG group, indicating that LPG improved B cell activation and their differentiation to memory B cells (Figure 3D, signaling panel). For transcriptional factors, the T_fh_ master transcriptional factor *BCL-6* and those responsible for B cell differentiation (*EBF1* and *PAX5*) (15) and the activation and switch to plasma cells (*XBP1)* (16) peaked on day 0 in the spleen and varied in LNs in the LPG group (Figure 3D, transcription panel). In general, the entire set of genes for B cell activation, proliferation, memory, and differentiation was up-regulated early in the LPG group, which corroborates our observation of improved SARS-CoV-2 humoral responses.

We further examined the transcriptional signatures of T cell subsets. We found that molecules of the CD28-B7, TNFSF/TNFRSF, and TRAF families were upregulated in the LPG group (Figure 3E, signaling panel), supporting our observation that LPG enhanced T cell responses to SARS-COV-2 (Figures 2F,G and Supplementary Figures 3A,B). Accordingly, IL genes and those of the down-stream JAK-STAT pathway were up-regulated in LPG group, including *IL-2R, IL-7R, IL-15R*, and *STAT1/3/5* (Figure 3E, signaling panel, middle). These data indicate that LPG could facilitate T cell survival and their differentiation into memory T cells. *CCR7, CXCR3, CXCR5*, and the *S1PR* family are associated with cell recruitment and antigen collection, and the decrease of *CCR7* and increase of *S1PR* family members in the spleen of the LPG group promoted T cell emigration from lymph tissues (Figure 3E, signaling panel, bottom). We further observed up-regulation of the master transcriptional factors such as *EOMES, TBX21, GATA3*, and *BCL6* in different CD4+ subsets, indicating a more active T cell response in the LPG group (17). We also found increased expression of *BATF, PRDM1*, and *IRF* family genes involved in sustaining virus-specific CD8+T cell function (18) (Figure 3E, transcriptional panel). The decreases in *IL-17A* and *IL-17B* indicate down-regulation of inflammation (19), while increases in *PRF1, GZMA*, and *GZMB* suggest vigorous cytotoxic function (20) (Figure 3E, cytokine panel) in the LPG group. Interestingly, all these observations manifested in the spleen and mesenteric LN, but not in the mediastinal LN, which may be due to the delayed response for the mediastinal LN as shown in Figure 3E.

## Discussion

It has been reported that antibody titers in subjects who have recovered from SARS-CoV-2 infection rapidly wanes over time (21). This raises concerns of how long vaccine-elicited immune responses will last, which will dictate when boost inoculation should be implemented. Importantly, both clinical trial and real-world data have shown that neutralization titers against SARS-CoV-2 (i.e., nAb values after adjustment with convalescent sera titers) correlate with protective efficacy (22), and breakthrough SARS-CoV-2 infection mainly occurs in subjects who receive vaccines with low nAb titers (23). For example, the regimen of rAd26-S plus rAd5-S elicited a 2-3-fold increase in nAb titers in comparison with ChAdOx1 nCoV-19 or Ad26.COV2.S. Correspondingly, the former conferred a 1.5-fold increase in protective efficacy, suggesting that a 2–3-fold difference in nAb titers is likely to significantly improve protection in the real world. It is therefore critical that licensed vaccines mount high, long-lasting nAb responses.

Here we demonstrated that the unique probiotic strain LBG boosted humoral responses against SARS-CoV-2 and stabilized RBD-specific T-cell responses in mice when given immediately after vaccination. We observed a >2-fold increase of nAb titers in LPG group sera and a >8-fold increase of nAb titers in LPG group BAL, which is more important than the increase in sera. The LPG group also exhibited enhanced T-cell responses, and as evidenced in Figure 1, LPG stabilized the memory responses for a prolonged time period. In an influenza vaccine clinical trial, administration of probiotic bacteria improved the proportion of nature killer cells and T-helper and T-cytotoxic cells. The greater immune response observed in the probiotic group in comparison with the placebo group correlated with a lower incidence of influenza-like illness in the 5 months of follow-up (24). Therefore, LGP may function as an adjuvant to enhance innate and adaptive immunity, thereby enhancing the protective efficacy and prolonging the protection from SARS-CoV-2 vaccination as observed for influenza and polio vaccination (4, 24–26).

Transcriptional data revealed that LPG alone could down-regulate inflammatory responses and the apoptotic pathway and up-regulate IFN responses in the absence of vaccination. As down-regulation of TNF-α and apoptosis pathways largely affects immune cells, oral LPG administration is likely to heavily imprint immune cell activation and differentiation to enhance immune cell survival and memory pool formation. Up-regulation of the IFN response pathway could facilitate the clearance of any local tissue pathogens and promote immune responses. All these observations support our hypothesis that LPG boosted and prolonged SARS-CoV-2 immune responses. Furthermore, LPG is capable of affecting gut-spleen and gut-lung immune regulatory axes to overcome the barrier between mucosal and systemic compartments and mobilize immune responses (27).

Several B cell function-associated genes such as *AKT1/2, AICDA*, and *EBI3* were up-regulated in the LPG group. It is known that antigen recognition by BCR initiates the BCR signaling cascade by phosphorylation of *CD79A* and *CD79B* (28), which engages the tyrosine kinases *SYK* and *LYN* and the adapter protein *BLNK*. B cell-intrinsic *AKT1/2* function is required for germinal center (GC) formation, maintenance, and plasma cell differentiation (29). *AICDA* is associated with somatic hypermutation and class switching (15). *EBI3* can promote B cell activation, and CD11c+ (*ITGAX* gene) B cells are mainly memory B cells (30, 31). All the factors mentioned above were up-regulated in the LPG group, suggesting that LPG could improve B cell activation and differentiation to memory B cells.

Three signals are associated with T cell activation: signal 1 “antigenic stimulation,” signal 2 “co-stimulatory signals through CD28 or 4-1BB,” and signal 3 “cytokines of IL-12, IL-21 or IFN-alpha.” In the current study, representative T-cell co-signal molecules of the CD28-B7, TNFSF/TNFRSF, and TRAF families (32, 33) were upregulated in the LPG group, as were survival-related cytokine genes (*IL-2R, IL-7R, IL-15R*, and *STAT1/3/5*) were up-regulated LPG group in this study. IL-2 is perhaps the earliest cytokine secreted by T cells following T cell receptor (TCR) stimulation (34), and it is important in the initiation of Th2 cell differentiation (35). It is well known that IL-2 can induce the proliferation and survival of TCR-activated human and mouse T cells (36) and is required for sustained expansion of T cell populations (37). IL-7 seems to be the most important γ_c_ family cytokine for regulating naïve and memory T cell homeostasis (38), while IL-15 is an important homeostatic cytokine with preferential activity for memory CD8+ T cells (39, 40). These data indicate that LPG could facilitate T cell survival and their differentiation into memory T cells.

Immune cell migration increases interaction between cells and is critical for the initiation of immune responses. In our study, the decrease of CCR7 and increase of S1PR family in the LPG group in the spleen promoted T cell emigration from lymph tissues. It has been speculated that emigration of CD8+ effector T cells from the T-zone may represent an important mechanism protecting professional antigen-presenting cells (APCs) against cytotoxic T cell attacks and preventing a premature decline in immune responses (41, 42), which may explain the prolonged T-cell specific response in the LPG group.

Overall, these transcriptional data demonstrated that oral administration of LPG is capable of enhancing gene expression associated with T cell and B cell activation, proliferation, survival, differentiation to memory cells, and migration. It is therefore rationalized that if co-administrated with vaccination, for example SARS-CoV-2 vaccine, LPG will enhance both immunogen-specific T and B cell responses, facilitate their mobilization among mucosal and systemic immune compartments, improve and prolong their memory responses.

A growing body of clinical data suggests that a cytokine storm is associated with COVID-19 severity and is a crucial cause of mortality (43, 44). IL-6 and TNF-α are both key pro-inflammatory cytokines, and their suppression may attenuate disease severity. Notably, one of the proposed predisposing factors for a cytokine storm is a low level of type I IFN (45). As described above, we observed a ~20-fold reduction of IL-6 and 3–8-fold decrease of TNF-α on day 3 and 7, respectively, in the BAL of the LPG group compared with the PBS group. The IFN response pathway was up-regulated in both local tissue and immune cells, so oral LPG is likely to attenuate respiratory inflammation and help contain SARS-CoV-2 replication, thereby ameliorating clinical manifestations and facilitate patient recovery.

In conclusion, our results demonstrated that LPG is able to boost vaccine-induced effective and memory immune responses by enhancing IFN signaling and suppressing apoptotic and inflammatory pathways. Co-administration of the SARS-CoV-2 vaccine and LPG show great potential to improve COVID-19 vaccination efficacy and contribute to the containment of the pandemic. Routine administration of LPG is likely to enhance the host innate immune responses to combat SARS-CoV-2.

## Data Availability Statement

The original contributions presented in the study are publicly available. This data can be found in the NCBI SRA database (accession numbers: 10520442, 10526585, 10614070).

## Ethics Statement

The animal study was reviewed and approved by Institutional Animal Care and Use Committee (IACUC) of Shanghai Public Health Clinical Center (Shanghai, China).

## Author Contributions

JGX conceived and designed the experiments. JQX, ZHR, and KLC designed and implemented the SARS-CoV-2 related experiments. XL, JL, DJ, TY, and LL prepared and carried out experiments. LZ and KLC analyzed the transcriptional data. XW assisted animal study and ELISA. LD performed neutralization antibody titers. All authors contributed to the article and approved the submitted version.

## Funding

This work was supported by National Key R and D Program of China (2019YFC1200501 and 2019 YFC 1200505), National Science and Technology Major Project of China (2018 Zx10712001-018), Research Units of Discovery of Unknown Bacteria and Function (2018RU010) to JGX, and Shanghai Municipal Science and Technology Commission (21S11903600), National Natural Science Foundation of China (81672018) to JQX.

## Conflict of Interest

JGX, JQX, ZHR, and KLC filed patent describing the invention and use of the probiotic described in the manuscript. The remaining authors declare that the research was conducted in the absence of any commercial or financial relationships that could be construed as a potential conflict of interest.

## Publisher's Note

All claims expressed in this article are solely those of the authors and do not necessarily represent those of their affiliated organizations, or those of the publisher, the editors and the reviewers. Any product that may be evaluated in this article, or claim that may be made by its manufacturer, is not guaranteed or endorsed by the publisher.

## Supplementary Material

Supplementary Figure 1 *Lactobacillus plantarum* GUANKE (LPG) genome analysis. The phylogenetic tree of *L. plantarum* (maximum-likelihood tree estimated using IQtree v.1.6.9) **(A)**. The phylogeny was alignment based on the SNP of concatenated core genes with the recombination region removed. The GUANKE strain in our study is marked in red. Genome comparison of *L. plantarum* GUANKE with *L. plantarum* str. J26 **(B)**. The inner second and third circles show the GC content and C/G skew of GUANKE. The outer circle shows the distribution and similarity of all ORFs in J26 compared to GUANKE with the missing genome annotated.

Supplementary Figure 2 Oral administration of LPG 6 months after immunization did not change the total IgG titer in mice. Sera were collected post intragastric administration for total IgG assessment by ELISA **(A)**, “Index” values are shown **(B)**, which represents the ratios of titers of binding antibodies at indicated time-points to their counterparts at baseline and reflects the adjusted relative values after the removal of differences at baseline.

Supplementary Figure 3 Oral LPG administration immediately after immunization stabilizes cellular immune responses in mice. Assessments of RBD-specific T cell responses. Splenocytes **(A)** and BAL cells **(B)** were isolated at day 3, 7, and 14 post intragastric administration and *in vitro* stimulated with 13 peptide pools (15-mer with 11 overlapped amino acids) covering the entire RBD sequence. The resulting IFN-γ-secreting cells were quantified by ELISpot. ELISpot counts are expressed as mean ± s.e.m. Mann-Whitney tests were performed to analyze differences between experimental groups. ^**^*P* < 0.01 and ns, not significant.

Supplementary Figure 4 Hierarchical cluster analysis of T and B cell profiles from the spleen, mesenteric lymph node, mediastinal lymph node, small intestine, and colon. Heatmap with hierarchical clustering analysis including all groups subjected to RNA-sequencing of the T cell- and B cell-associated genes, with relative expression of log2 RPKM.
