# Supplementary figures and images for: Boosting Vaccine-Elicited Respiratory Mucosal and Systemic COVID-19 Immunity in Mice With the Oral Lactobacillus plantarum
Source: Front Nutr. 2021 Dec 22;8:789242. doi: 10.3389/fnut.2021.789242 (PMC8733898; doi:10.3389/fnut.2021.789242)

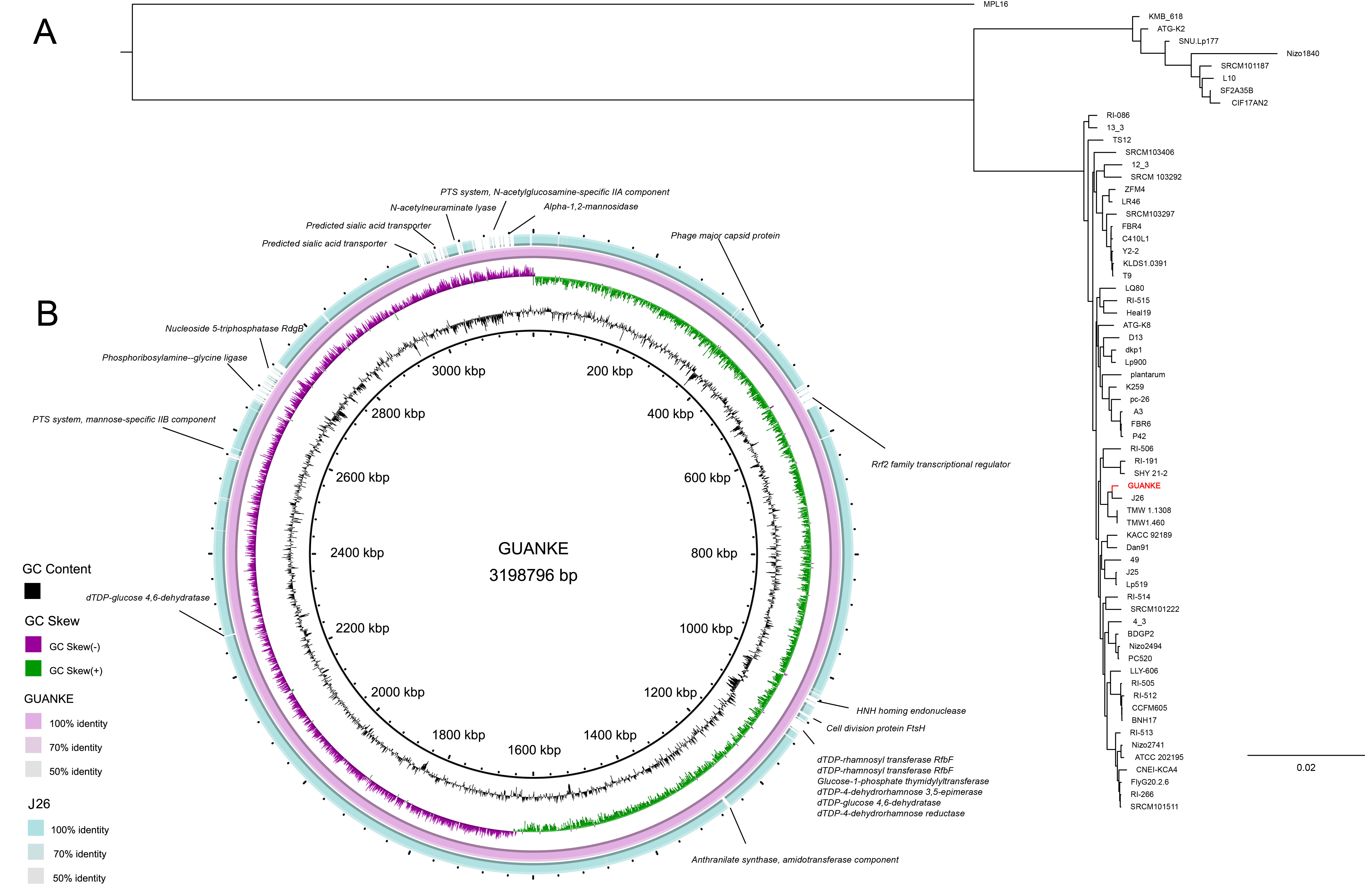

Supplement: Supplementary Figure 1 — Lactobacillus plantarum GUANKE (LPG) genome analysis. The phylogenetic tree of L. plantarum (maximum-likelihood tree estimated using IQtree v.1.6.9) (A). The phylogeny was alignment based on the SNP of concatenated core genes with the recombination region removed. The GUANKE strain in our study is marked in red. Genome comparison of L. plantarum GUANKE with L. plantarum str. J26 (B). The inner second and third circles show the GC content and C/G skew of GUANKE. The outer circle shows the distribution and similarity of all ORFs in J26 compared to GUANKE with the missing genome annotated. [file Image_1.tif]

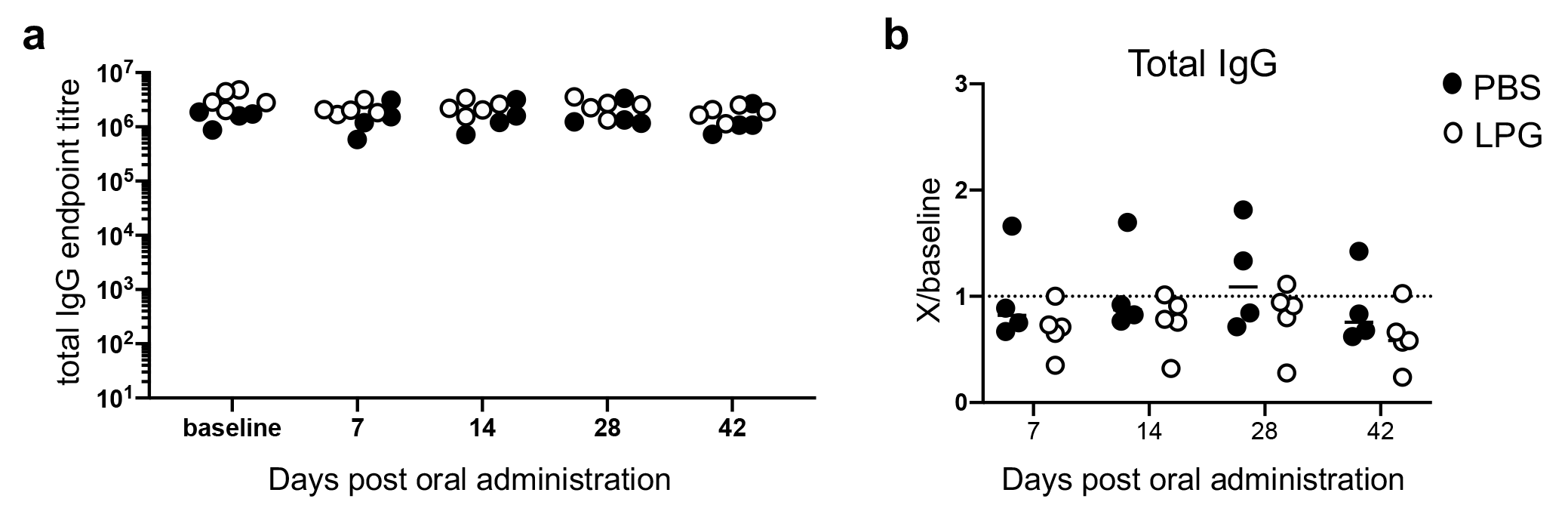

Supplement: Supplementary Figure 2 — Oral administration of LPG 6 months after immunization did not change the total IgG titer in mice. Sera were collected post intragastric administration for total IgG assessment by ELISA (A), “Index” values are shown (B), which represents the ratios of titers of binding antibodies at indicated time-points to their counterparts at baseline and reflects the adjusted relative values after the removal of differences at baseline. [file Image_2.tif]

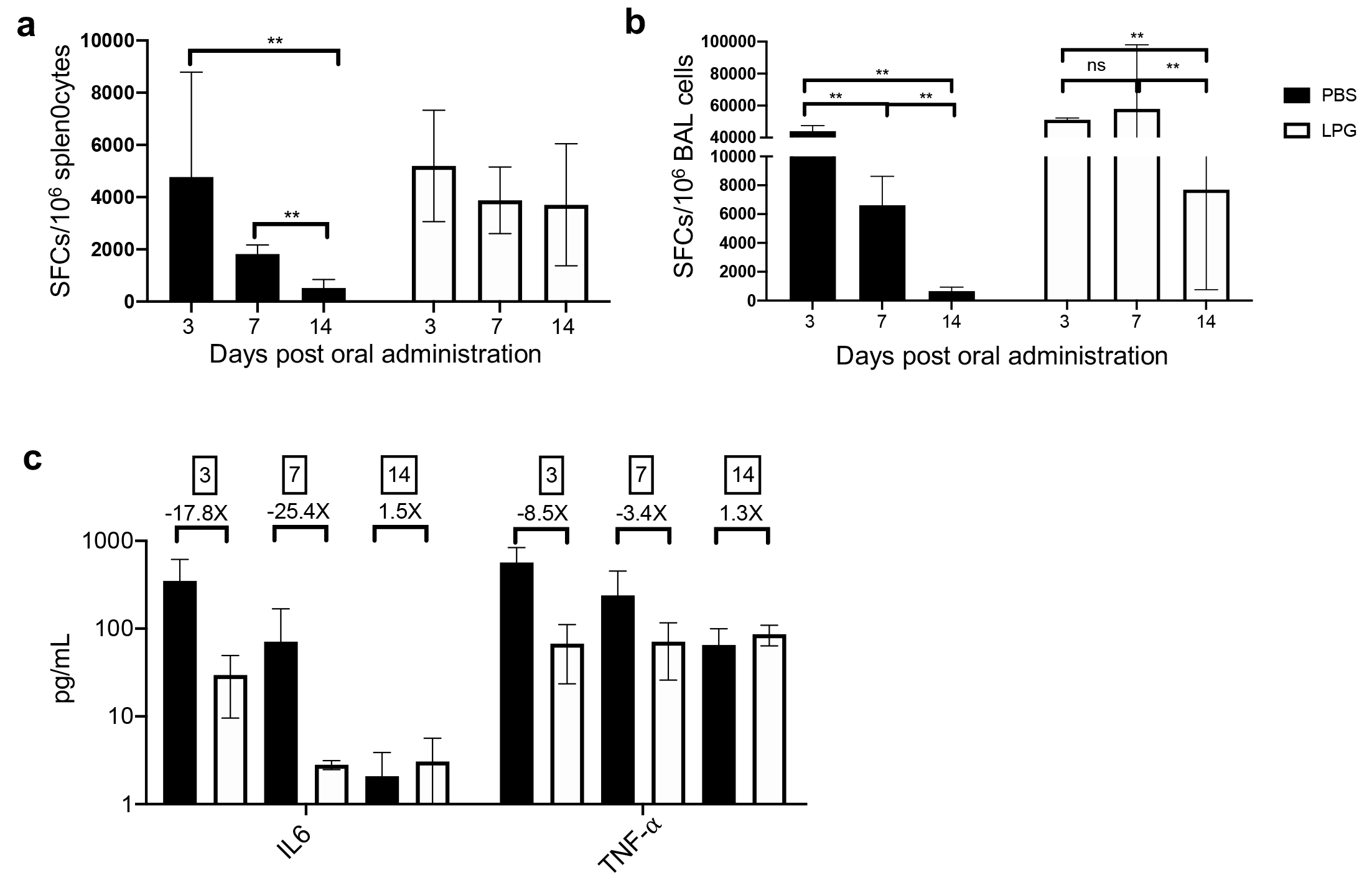

Supplement: Supplementary Figure 3 — Oral LPG administration immediately after immunization stabilizes cellular immune responses in mice. Assessments of RBD-specific T cell responses. Splenocytes (A) and BAL cells (B) were isolated at day 3, 7, and 14 post intragastric administration and in vitro stimulated with 13 peptide pools (15-mer with 11 overlapped amino acids) covering the entire RBD sequence. The resulting IFN-γ-secreting cells were quantified by ELISpot. ELISpot counts are expressed as mean ± s.e.m. Mann-Whitney tests were performed to analyze differences between experimental groups. **P < 0.01 and ns, not significant. [file Image_3.tif]

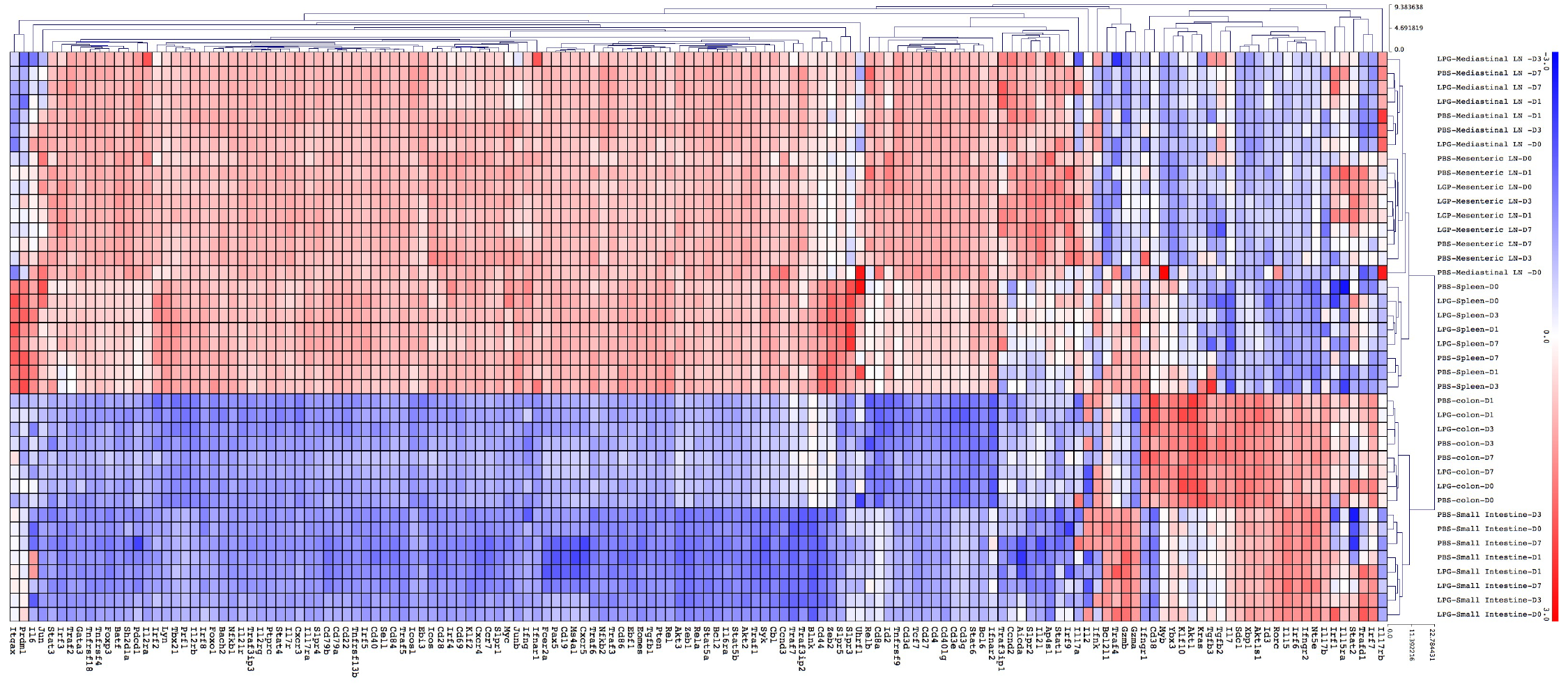

Supplement: Supplementary Figure 4 — Hierarchical cluster analysis of T and B cell profiles from the spleen, mesenteric lymph node, mediastinal lymph node, small intestine, and colon. Heatmap with hierarchical clustering analysis including all groups subjected to RNA-sequencing of the T cell- and B cell-associated genes, with relative expression of log2 RPKM. [file Image_4.tif]
